# Does sex interact with the 2D:4D ratio or adult circulating hormones on the estimated glomerular filtration rate? A cross‐sectional study in Ghana

**DOI:** 10.14814/phy2.15516

**Published:** 2022-11-16

**Authors:** Moses Banyeh, Kervin Edinam Zogli, Hisham Alhassan Osumanu, Lawrence Obeng, Tuah Kwabena Acheampong, Clement Binwatin Dagungong, Elizabeth Bayor, Nafiu Amidu

**Affiliations:** ^1^ Department of Biomedical Laboratory Science University for Development Studies Tamale Ghana

**Keywords:** adult, Ghana, glomerular filtration rate, humans, the 2D:4D ratio

## Abstract

The 2D:4D ratio is the putative marker of prenatal hormone exposure and has been suggested as a correlate of adult circulating testosterone and estrogen. The study aimed to determine whether sexual dimorphism in the estimated glomerular filtration rate (eGFR) can be partly explained by the 2D:4D ratio or adult circulating testosterone or estrogen. The study was cross‐sectional from June to December 2021 at the University for Development Studies. The study involved 206 healthy adults (Female = 93, Male = 113) between 18 and 30 years. The 2D:4D ratio was measured using computer‐assisted analysis. Venous blood samples were collected and analyzed for testosterone, estradiol and creatinine using the ELISA technique and routine biochemical analysis. The adjusted eGFR was calculated using the Chronic Kidney Disease Epidemiology Collaboration (CKD‐EPI) creatinine equation (2021). The eGFR and the testosterone‐to‐estradiol ratio (TT:E_2_) were significantly higher in males than in females (*p* < 0.001). There was a significant interaction between sex and the TT:E_2_ on the eGFR (*p* < 0.001). Although the relationship between the eGFR and the TT:E_2_ was negative in both males and females, a unit change in the TT:E_2_ had a greater impact on the eGFR in females (*B* = −1.38) than in males (*B* = −0.01). Sexual dimorphism in the eGFR is influenced by both testosterone and estradiol. Although the sex difference in the eGFR may be influenced by the TT:E_2_, estrogen seems to account for more variability in the eGFR than testosterone.

## Graphical abstract

The testosterone‐to‐estradiol ratio may partly account for the sexual dimorphism in the estimated glomerular filtration rate among healthy adults.

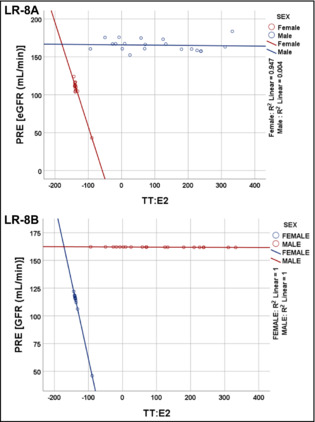

## 1 INTRODUCTION

There is sexual dimorphism in renal function in both health and disease. In general, men have a relatively higher prevalence and incidence of end‐stage renal disease (ESRD) than women although women have a greater odds of chronic kidney disease (CKD) than similarly aged men. This demonstrates that the progression of renal disease may be faster in men than in women (Bairey Merz et al., 2019; Goldberg & Krause, 2016; Neugarten et al., 2000). However, men may have a comparatively higher renal functional reserve that may compensate for the accumulation of sclerotic glomeruli due to the effect of aging. Findings from previous studies show that healthy men may have a greater capacity to maintain their glomerular filtration rate (GFR) by increasing the filtration fraction than women (Xu et al., 2010). While the number of nephrons is similar in males and females, in general, males have a higher kidney mass than females. Also, there is hypertrophy in the cortical proximal tubular cells of the male kidney relative to females which may partly explain the sexual dimorphism in renal function (Hosszu et al., 2020; Sabolić et al., 2007). Previous studies had observed that women may have a relatively higher renovascular resistance, coupled with a lower absolute GFR, and a relatively lower renal plasma flow than men (Hosszu et al., 2020). Sex differences in renal function and renal disease prevalence have been attributed to the activities of sex hormones such as testosterone and estrogen (Bairey Merz et al., 2019; Goldberg & Krause, 2016; Neugarten et al., 2000).

The administration of sex hormones or gonadectomy has also provided pieces of evidence in support of the role of sex hormones in kidney health (Neugarten et al., 2000). Sex hormones influence the development of specific traits among males and females and this primarily affects the structure and function of the kidneys and the renal system (Sabolić et al., 2007). Some data from previous studies sought to suggest that estrogen may be reno‐protective whereas testosterone may either be a ‘bystander’ or detrimental to renal function (Silbiger, 2011). Evidence of this observation has been seen in the reduced prevalence of CKD and slower progression of renal disease to ESRD in younger than older women. Also, the incidence of CKD is relatively higher among postmenopausal women or premenopausal women who underwent oophorectomy (Grewal & Blake, 2005). But studies have also shown that the exogenous administration of estrogen does not necessarily improve GFR, renal blood flow or renal disease prognosis (Bairey Merz et al., 2019).

It is also worth noting that sexual dimorphism in renal function should be interpreted against the method used in estimating the GFR as well as ethnicity. There have been variabilities in published equations for estimating GFR in different populations and opinions are varied on whether such equations should be adjusted for ethnicity or not (Holness et al., 2020; Rocha et al., 2020). Also, the online source of the GFR calculator should be verified as available online calculators have been found to vary in their estimation of the GFR (Seiberth et al., 2020). The most common estimating equations for GFR include the Chronic Kidney Disease Epidemiology Collaboration (CKD‐EPI), The Modification of Diet in Renal Disease (MDRD), the Cockcroft‐Gault (CG), and the full age spectrum (FAS) (Omuse et al., 2017).

According to the Organizational Hypothesis, the early exposure in life, to androgens e.g. testosterone, permanently musculinizes the brain and behavior (Breedlove, 2010). Also, prenatal testosterone (PT) exposure may lead to sexual dimorphism in human phenotypic traits including digit length ratios (Breedlove, 2010; Zheng & Cohn, 2011). The second‐to‐fourth digit ratio (2D:4D) is the putative marker of PT exposure. Previous studies have shown that the 2D:4D ratio is not affected by exposure to only PT but by a balanced exposure to both PT and prenatal estrogen (PE) during a narrow window in prenatal development (Zheng & Cohn, 2011). The 2D:4D ratio and the right–left difference (Dr‐l) are similar in pattern because a low 2D:4D of the right hand may be associated with more masculine traits while a high ratio may be associated with feminine traits. The 2D:4D or the Dr‐l are negative and positive correlates of PT and PE exposure respectively and are lower in females than males on average (Manning, 2002). They have also been found to correlate positively with circulating testosterone but negatively with circulating estrogen in adulthood, although this observation has not been universal (Hönekopp et al., 2007; Manning, 2002; Muller et al., 2011; Richards et al., 2018). Also, the use of the 2D:4D ratio or the Dr‐l as putative markers of PT and PE exposure has been controversial (Leslie, 2019). However, an overview and a critical review of the available literature have provided pieces of evidence in support of their validity (McCormick & Carré, 2020; Swift‐Gallant et al., 2020). Interestingly, the Homeobox box (Hox genes) family of genes partly control both digits development and urethra‐genital differentiation (Manning et al., 2003). Given the above, it is plausible to suggest that renal function in healthy adults may be associated with the 2D:4D, adult circulating hormones or both.

A review of the available literature has shown no known study that has examined the possible association between the estimated glomerular filtration rate (eGFR) and both the 2D:4D ratio and circulating hormones in a healthy adult population. This study, therefore, sought to determine whether sex interacts with the 2D:4D ratio or circulating testosterone, estradiol or their derivatives on the eGFR in a healthy Ghanaian population.

## 2 MATERIALS AND METHODS

### 2.1 Study design and setting

The study was cross‐sectional from June to December 2021 at the Tamale campus of the University for Development Studies (UDS). The University for Development Studies is a public tertiary institution that is located in the Northern Region of Ghana. (UDS, 2020).

### 2.2 Study population

The study participants were 206 (Female = 93, Male = 113) and were aged between 18 and 30 years. The study participants were part of a larger study from which a paper has already been published (Banyeh et al., 2022). All the participants had no known history of fractures that could markedly affect finger length and height measurement. Participants who had a known history of chronic kidney disease (CKD) or any kidney‐related condition were excluded. Also, participants with a known history of hormonal abnormalities or females on hormonal contraceptives were excluded from the study. The study was voluntary without limitations regarding religious, cultural or political affiliations.

### 2.3 Variables

The dependent variable was the estimated glomerular filtration rate (eGFR) which was calculated using serum creatinine (sCRT), the age at sampling in years, the standing height in centimeters and body weight in Kilogram. The independent variables were: the right hand (2D:4DR), the left hand (2D:4DL) digit ratio and the right–left hand difference (Dr‐l); total testosterone (TT), estradiol (E_2_) and their calculated indices, free testosterone (FT), bioavailable testosterone (BioT), the free androgen index (FAI) and the total testosterone‐to‐estradiol ratio (TT/E_2_).

### 2.4 Measurements

**FIGURE 1:**
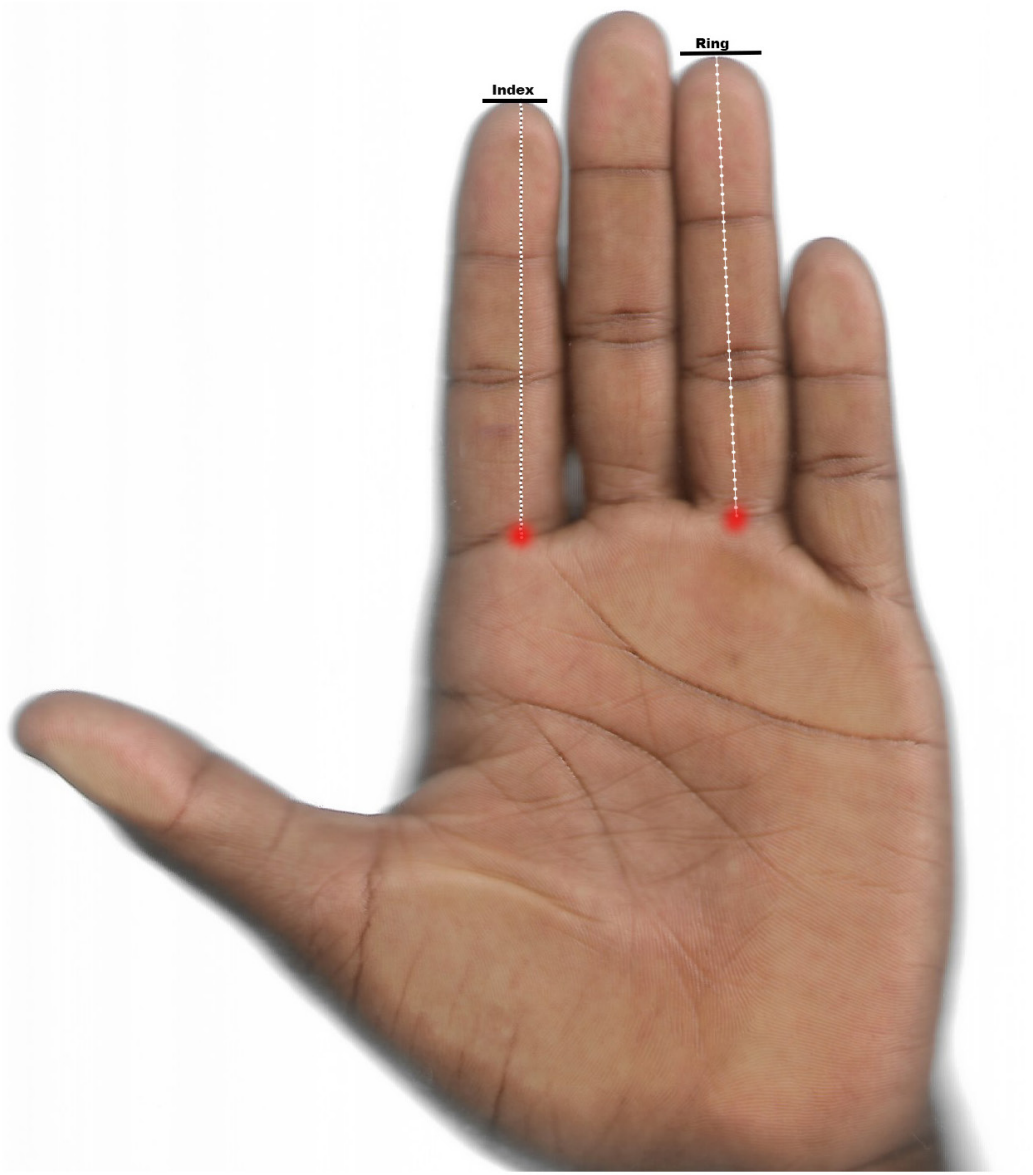
The scanned image of the palmar surface of the hand. The length of the second and fourth fingers was measured from the most proximal crease to the tip of the finger.

The standing height (cm) and body weight (kg) were measured using a stadiometer and a bathroom scale and following the recommended guidelines (Gualdi‐Russo et al., 2018). The palmar surface of the right and left hands of each participant were scanned alongside their unique study identification numbers using the Hp desk jet 2620 all‐in‐one printer scanner (Figure 1). The scanned images were exported to a computer program [GIMP, (v 2.10.22), www.gimp.org]. The lengths of the second and fourth digits were then measured twice from the hand scans by one observer (at a week's intervals) using the digital caliper on the computer screen to the nearest 0.01 millimeter. The finger length was measured from the most proximal palmar crease to the tip of the finger (Manning et al., 1998). The intraclass correlation coefficients (ICC) between the repeated measurements were calculated using the two‐way mixed, single measures with absolute agreement technique. The ICC were 0.950 and 0.966 for the 2D:4DR and 2D:4DL respectively. The right (2D:4DR) and the left (2D:4DL) ratios were then calculated as the ratio of the second‐to‐fourth digit. The right–left 2D:4D difference (Dr‐l) was also calculated. A single venous blood sample was collected after an overnight fast (8‐14 h) into a gel separator vacutainer tube and stored at 4°C. The blood samples were allowed to clot before they were centrifuged at 1500 rpm for 10 min to obtain serum. All sampling was done between 8‐ and 12 h of Greenwich Mean Time (GMT) to reduce diurnal variabilities. The serum samples were then aliquoted and stored at ‐25°C for analysis. The serum samples were assayed for total testosterone, estradiol and sex hormone‐binding globulin (SHBG) using ELISA test kits (Monobind Inc.). The free testosterone (FT%) and bioavailable testosterone (BioT%) were calculated from the website (http://www.issam.ch/freetesto.html) based on the recommended formula by Vermeulen, et al. (Vermeulen et al., 1999). The free androgen index (FAI) and testosterone‐to‐estradiol ratio (TT/E_2_) were calculated by dividing TT by SHBG and E_2_ respectively (all in the same unit). The plasma/serum creatinine was measured using routine biochemistry on the BT 1500 automated biochemistry analyzer (Biotechnica Instruments, SPA) following the manufacturer's instructions and using the recommended reagents. The plasma/serum samples were not previously thawed and refrozen. The estimated glomerular filtration rate (eGFR) was calculated based on the Chronic Kidney Disease Epidemiology Collaboration (CKD‐EPI) creatinine equation (2021) of the National Kidney Foundation [https://www.kidney.org/professionals/KDOQI/gfr_calculator]. The eGFR was adjusted for age and the body surface area including the standing height (cm) and body weight (Kg):

$$\begin{array}{c} {\text{eGFR}}_{\mathrm{cr}}\;\left(\mathrm{ml}/\min\right)=142\;\times \;\min\;{\left(S_{\mathrm{cr}}/\kappa ,,,1\right)}^{\alpha }\\ \;\times \;\max{\left(S_{\mathrm{cr}}/\kappa ,,,1\right)}^{-1.200}\;\times \;0.9938^{\mathrm{Age}}\\ \;\times \;1.012\;\left[\text{if female}\right]. \end{array}$$

where:

S_cr_ = serum creatinine in mg/dL.

κ = 0.7 (females) or 0.9 (males).

α = −0.241 (female) or −0.302 (male).

min (S_cr_/κ, 1) is the minimum of S_cr_/κ or 1.0.

max (S_cr_/κ, 1) is the maximum of S_cr_/κ or 1.0.

Age (years).

### 2.5 Bias mitigation

To reduce bias, the estimated glomerular filtration rate (eGFR) was adjusted for body surface area by adding the standing height (cm) and the body weight (kg) to the equation. And as such the linear regression models were not adjusted for age and BMI since that may amount to over‐adjustment of the models.

### 2.6 Statistical analysis

The data were collected and sorted in an Excel Spreadsheet before statistical analysis in SPSS (v26) and GraphPad Prism (v8). Where missing values were less than 5%, those missing values were replaced with the series mean, separately for males and females. The normality of the data was checked using the Shapiro–Wilk Test. Descriptive statistics were performed and the results were presented as mean ± standard deviation (SD) or median (interquartile range‐IQR) for parametric and non‐parametric variables. The differences between male and female mean or median values were tested using the student *t*‐test or the Mann–Whitney U test (unpaired, 2‐tailed) respectively. Asymmetry in the right–left hand 2D:4D ratio (Dr‐l) was compared to zero (0) using the one sample, one‐tailed *t*‐test. To avoid or reduce multicollinearity, the independent variables were centered on their means by subtracting the mean value from the variable. Two‐way interaction terms were then created between the centered variable and the sex (coded: female = 0, male = 1) variable (e.g. SEX*2D:4DR‐centered). Linear regression models were formulated with the eGFR as the dependent variables and the sex, centered variable and the interaction term as the predictor variables. The regression models were not adjusted for age and BMI since age, height and weight were included in calculating the eGFR. The assumptions of linear regression were tested using the variance inflation factor (VIF) for multicollinearity, the Durbin‐Watson test for autocorrelation and the Cook's distance (Cook's D) for influential multivariable outliers. Also, the standardized regression residuals and predicted values were used to test for multivariable normality using a histogram and the probability‐probability plot (P–P plot) and a scatterplot was used to test for homoscedasticity. To display the interaction effect graphically, the unstandardized predicted value of the dependent variable (eGFR) was plotted on the *y*‐axis against the predictor variable with sex as the marking variable. Where the assumption of homoscedasticity was violated for a selected model, weighted regression analysis was also performed. A weight was created from the regression residuals using auxiliary regression analysis and was then included in the new model. All the statistical analyses were considered significant at *p* < 0.050.

### 2.7 Ethical declaration

The authors complied with the guidelines regarding human subject studies as contained in the 1964 Declaration of Helsinki and its later amendments. The study was approved by the institutional review board of the University for Development Studies (N#: UDS/RB/003/21). Written informed consent was obtained from each participant before the study.

## 3 RESULTS

### 3.1 General characteristics of the study population

The sex differences in variables of the study population are summarized in Table 1. The males of the study population were significantly taller but had lower BMI than females (*p* < 0.050). Also, males had significantly higher TT, TT:E_2_, and eGFR than females (*p* < 0.050). However, no sex difference in the 2D:4D ratio was observed.

**TABLE 1:** General characteristics of the study population

Variable	Female	Male	*p*‐value
Weight (kg)	61.5 ± 11.1	65.4 ± 9.5	0.062
Height (cm)	162 ± 6.9	173 ± 7.3	<0.001
BMI (kg/m^2^)	23.4 ± 3.7	21.8 ± 2.6	0.017
sCRT (μmol/L)	61.5 (48.7–82.4)	77.2 (57.2–98.9)	0.026
eGFR (ml/min)	107.8 ± 25.7	165.5 ± 39.5	<0.001
TT (nmol/L)	1.27 ± 0.30	21.70 ± 4.73	<0.001
FT (%)	0.63 ± 0.18	1.35 ± 0.41	<0.001
BioT(%)	14.55 ± 4.37	31.56 ± 9.60	<0.001
FAI	0.001 ± 0.004	0.47 ± 0.25	<0.001
E_2_ (pg/ml)	71.53 ± 22.91	29.43 ± 7.45	<0.001
TT:E_2_	8.65 ± 6.91	245.83 ± 76.26	<0.001
SHBG (nmol/L)	187.31 ± 61.78	82.59 ± 42.77	<0.001
2D:4DR	0.937 ± 0.040	0.935 ± 0.036	0.792
2D:4DL	0.940 ± 0.040	0.935 ± 0.034	0.498
Dr‐l	−0.003 ± 0.027	−0.000 ± 0.028	0.590

Abbreviations: 2D:4D, second‐to‐fourth digit ratio; BioT, bioavailable testosterone; BMI, body mass index; Dr‐l, right–left 2D:4D; E_2_, estradiol; eGFR, estimated glomerular filtration rate; FAI, free androgen index; FT, free testosterone; L, lefthand; R, righthand; sCRT, serum creatinine; SHBG, sex‐hormone binding globulin; TT, total testosterone.

### 3.2 Linear regression with interaction terms

**FIGURE 2:**
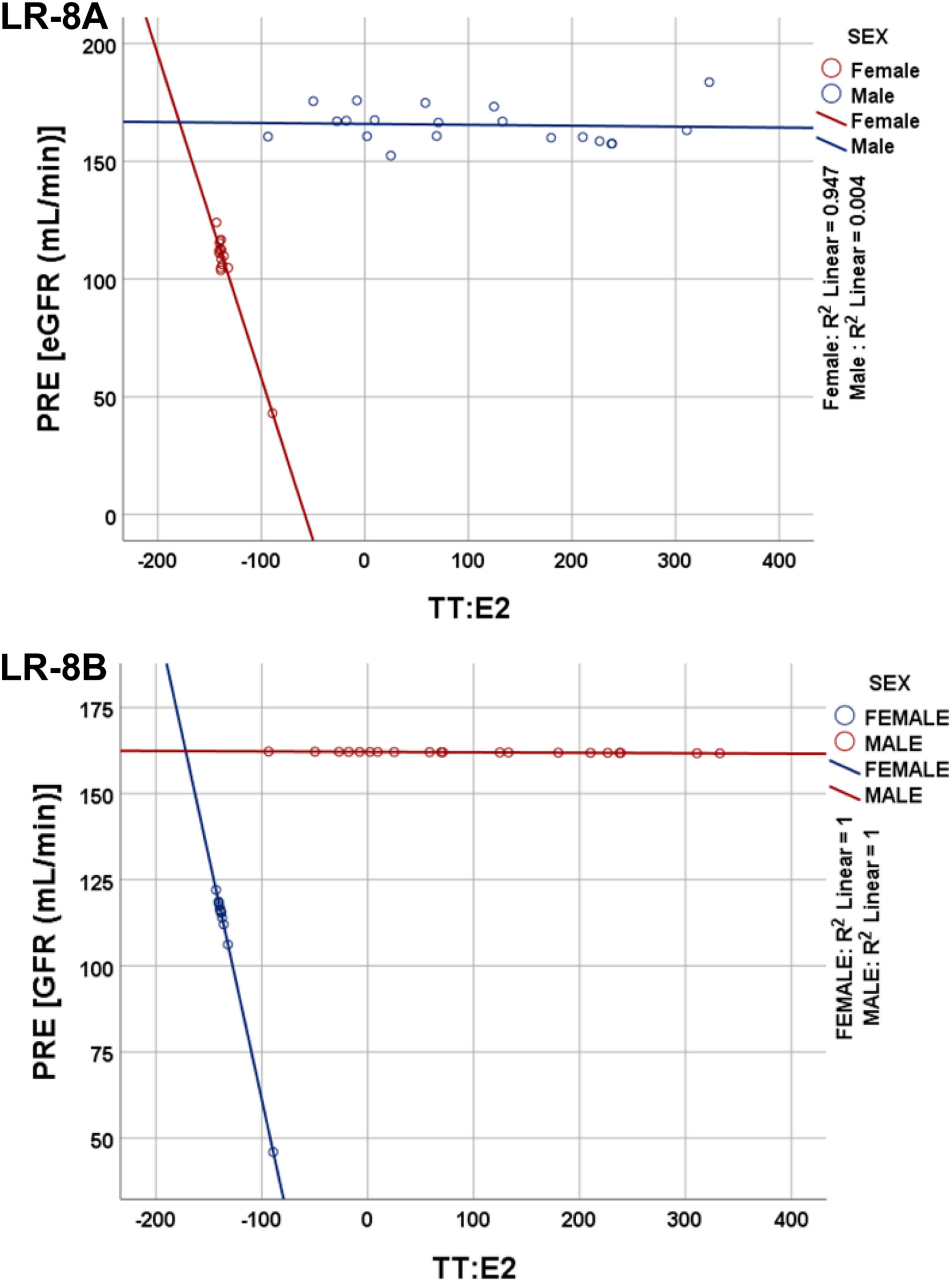
The impact of the testosterone‐to‐estradiol ratio (TT:E_2_) on sexual dimorphism in the estimated glomerular filtration rate (eGFR). The unstandardized predicted values of the dependent variable (PRE) were plotted against the predictor variable (TT:E_2_) while sex was used as the marking variable. Models were formulated without a weight (LR‐8A) and with a weight (LR‐8B).

The sex‐moderated linear regression models showing the relationship between eGFR and the independent variables of the study population are summarized in Tables 2 and 3 and Figure 2. The assumptions of multivariable linear regression were tested for LR‐8A. The assumption of homoscedasticity was violated (Supplementary Figure S1C). However, the variance inflation factor were from 1.11–2.92 (Ref: <10), the Cook's distance ranged from 0.00–0.12 (Ref: <1.00) while the Durbin‐Watson value was 1.65 (Ref: 50–2.50; Noel et al., 2021; Tranmer & Elliot, 2008). A weighted regression analysis was therefore performed (LR model 8B). In general, there was significant sexual dimorphism in the eGFR in LR models (*p* < 0.050). Also, there was a significant interaction between sex and the TT:E_2_ on the eGFR (*p* < 0.001). From the regression equations below, a unit change in TT:E_2_ resulted in about 1.35 ml/min (Equation 2) and about 0.01 ml/min (Equation 3) reduction in eGFR in females and males, respectively.

**TABLE 2:** Relationship between the eGFR and the ratio variables of the second and fourth digits

LR model	eGFR (ml/min)	*B*	95% CI		*p*‐value
			Lower	Upper	
1	**(Constant)**	107.64	98.42	116.86	<0.001
	SEX	57.89	44.72	71.06	<0.001
	2D:4DR	172.61	−59.38	404.60	0.143
	SEX*2D:4DR	−149.17	−498.20	199.86	0.398
2	**(Constant)**	107.52	98.41	116.63	<0.001
	SEX	57.71	44.65	70.76	<0.001
	2D:4DL	240.53	9.20	471.87	0.042
	SEX*2D:4DL	−317.48	−677.33	42.37	0.083
3	**(Constant)**	107.68	98.42	116.95	<0.001
	SEX	57.55	44.31	70.79	<0.001
	Dr‐l	−136.09	−476.96	204.77	0.430
	SEX* Dr‐l	286.97	−192.85	766.79	0.238

*Note*: Multivariable linear regression (LR) analysis with 2‐way interaction terms. The 2D:4D were centered on their means to reduce multicollinearity. The models were not adjusted for age and BMI because the estimated glomerular filtration rate (eGFR) was adjusted for age (years), height (cm) and weight (kg).

Abbreviations: CI, confidence interval; L, left hand; R, right hand.

**TABLE 3:** The relationship between the eGFR and adult circulating hormonal variables

LR model	eGFR (ml/min)	*B*	95% CI		*p*‐value
			Lower	Upper	
4	**(Constant)**	−77.70	−391.32	235.93	0.624
	SEX	235.89	−78.58	550.35	0.140
	TT (nmol/L)	−18.53	−49.85	12.78	0.243
	SEX*TT	19.24	−12.14	50.61	0.227
5	**(Constant)**	108.69	88.08	129.31	<0.001
	SEX	57.64	33.37	81.91	<0.001
	FT (%)	2.44	−50.35	55.23	0.927
	SEX*FT	−4.69	−62.46	53.08	0.872
6	**(Constant)**	108.48	88.26	128.71	<0.001
	SEX	57.86	33.88	81.84	<0.001
	BioT (%)	0.08	−2.08	2.23	0.944
	SEX*BioT	−0.17	−2.55	2.20	0.885
7	**(Constant)**	107.84	98.60	117.09	<0.001
	SEX	60.97	45.01	76.92	<0.001
	SEX*FAI	−13.87	−51.48	23.75	0.466
8A	**(Constant)**	−51.98	−207.21	103.24	0.508
	SEX	217.96	61.80	374.13	0.007
	TT:E_2_	−1.38	−2.71	−0.04	0.043
	SEX*TT:E_2_	1.37	0.03	2.71	0.045
8B	**(Constant)**	−48.67	−104.40	7.07	0.086
	SEX	214.72	155.18	274.27	<0.001
	TT/E2	−1.35	−1.84	−0.86	<0.001
	SEX*TT:E_2_	1.34	0.83	1.85	<0.001
9	**(Constant)**	105.91	93.36	118.45	<0.001
	SEX	70.57	38.85	102.30	<0.001
	E2 (pg/ml)	0.09	−0.32	0.50	0.650
	SEX*E_2_	0.42	−0.93	1.76	0.541

*Note*: Multivariable linear regression (LR) analysis with 2‐way interaction terms. The hormonal variables were centered on their means to reduce multicollinearity. The models were not adjusted for age and BMI because age, height, and weight were included in the calculation of the estimated glomerular filtration rate (eGFR).

Abbreviations: BioT, bioavailable testosterone; C, confidence interval; E2, estradiol; FAI, free androgen index; FT, free testosterone; TT, total testosterone; TT/E2, total testosterone‐to‐estradiol ratio.

*LR Model 8B*:

(1) $$\begin{array}{c} \text{eGFR}\;\left(\mathrm{ml}/\min\right)=-48.67+214.72^{*}\mathrm{SEX}–\;1.35^{*}\mathrm{TT}\\ \;/\text{E2}+1.34\;\left({\mathrm{SEX}}^{*}\mathrm{TT}/\text{E2}\right), \end{array}$$

(2) $$\begin{array}{c} \text{Female}\;\left(\text{code}=0\right):\text{eGFR}=-\;48.67–1.35^{*}\mathrm{TT}/\text{E2}, \end{array}$$

(3) $$\text{Male}\;\left(\text{code}=1\right):\text{eGFR}=166.05–0.01^{*}\mathrm{TT}/\text{E2}.$$

## 4 DISCUSSION

The study aimed to determine whether the 2D:4D ratio or adult circulating hormones interact with sex on the eGFR among healthy adults. It was observed that males had significantly higher total testosterone and the testosterone‐to‐estradiol ratio than females while the reverse was true for estradiol. There was sexual dimorphism in the eGFR in the study population where males had higher eGFR than females. Also, there was a significant interaction between sex and the TT:E_2_ on the eGFR. Although the effect of TT:E_2_ on eGFR was negative in both males and females, the impact of TT:E_2_ on the eGFR was more marked in females than males. There was no significant interaction between sex and the 2D:4D ratio on adult eGFR in this study.

The observation of higher testosterone or the testosterone‐to‐estradiol ratio in males than females is largely due to sex differences in testosterone production. Testosterone production begins in the testicular Leydig cells of males between the 6th and 7th weeks of gestation and peaking around the 13th‐14th week (O'shaughnessy et al., 2006). In females, the adrenal gland and the ovaries account for about half of all the testosterone production while the other half is produced from bones, fat, muscle and other peripheral tissues. Testosterone production in males is about 2.5 times that of females between gestational weeks 8 and 24 (Knickmeyer et al., 2005). If the activity of foetal Leydig activity is correlated with that in adulthood, then males would have significantly higher circulating testosterone than similarly‐aged females (Manning et al., 1998).

In this study, eGFR in males was significantly higher than in females. This finding is consistent with a previous study (Rule et al., 2004). Men may have a comparatively higher renal functional reserve that may compensate for the accumulation of sclerotic glomeruli due to the effect of aging. Findings from previous studies show that healthy men may have a greater capacity to maintain their glomerular filtration rate (GFRs) by increasing the filtration fraction than women(Xu et al., 2010). However, some previous studies found that females had higher eGFR than males while in others, no significant differences were observed between males and females (Cohen et al., 2014; Grewal & Blake, 2005; Xu et al., 2010; Yue et al., 2021). The differences between this and previous studies may be due to differences in genetic and environmental factors between populations. One important factor that may also account for variabilities between study results in the eGFR may be methodological. Opinions are varied regarding the use of the CKD‐EPI creatinine equation (2021) in the estimation of the eGFR. While some authors suggest the equation should be adjusted for ethnicity, particularly for persons of Black African ancestry, others do not find it necessary (Rocha et al., 2020). Some argue that the recommendation for a correction factor in the CKD‐EPI equation was extrapolated from African‐Americans to Black Africans which may not be justified. However, the CKD‐EPI creatinine equation was found to be comparable to the MDRD among mixed‐ancestry South Africans and even outperformed other equations when it was tested in healthy Black African and Brazilian Black populations without adjusting for ancestry (Holness et al., 2020; Moodley et al., 2018; Omuse et al., 2017; Rocha et al., 2020). Moreover the CKD‐EPI equation has been validated in the Ghanaian population in a previous study by Eastwood, et al. (Eastwood et al., 2010) and was found to be suitable for measuring the eGFR.

There is sexual dimorphism in eGFR among healthy adults. If the impact of TT:E_2_ on the eGFR is more in females than males, then adult circulating estrogen may have more impact on the eGFR than testosterone since females have lower testosterone but higher estrogen than males on average. This may support the observation that testosterone may just have a “bystander” effect on the eGFR as compared to estrogen (Garovic & August, 2016; Silbiger, 2011). The effect of estrogen on renal function is mediated by the estrogen receptors. Both the alpha (ER‐α) and beta (ER‐β) estrogen receptors have been found in the kidney, at least in experimental animals (Bairey Merz et al., 2019; Sabolić et al., 2007; Silbiger, 2011).

Estrogen is reno‐protective due to the delayed development of ESRD in women as compared to men. Some authors have suggested that the reno‐protective property of estrogen is through the inhibition of growth factor‐β‐induced collagen synthesis (Cohen et al., 2014; Silbiger, 2011). Estrogen may interact with the renin‐angiotensin system (RAS), endothelin‐1 and enzyme expression to influence renal function. Estrogen tends to induce the expression of the angiotensin type 2 receptors and increase the activity of nitric oxide synthase, leading to the release of more nitric oxide, a vasodilator but tends to inhibit the vasoconstrictive effect of endothelin‐1. Estrogen has also been demonstrated to have antioxidant activity by suppressing NADPH oxidase activity which tends to protect the renal system against the deleterious effect of reactive oxygen species (Cohen et al., 2014; Neugarten et al., 2000). Moreover, estrogen has an anti‐apoptotic and proliferative effect on the proximal renal tubular cells across the various phases of the menstrual cycle that are characterized by high estrogen levels. This observation has been corroborated by the higher GFR in pregnant women compared to their similarly aged non‐pregnant counterparts (Garovic & August, 2016). Estrogen has also been suggested to be responsible for the higher renovascular resistance in females which may protect the glomerulus from hyperfiltration‐induced injury by maintaining stability in the glomerular blood pressure (Bairey Merz et al., 2019). The kidney also performs a major role in maintaining the homeostasis of body fluid and electrolytes acting through localized transport proteins on the nephrons' apical and basolateral membrane domains. This function of the kidney may also be influenced by sex hormones and particularly estrogen (Bairey Merz et al., 2019; Sabolić et al., 2007).

The 2D:4D ratio was not significantly associated with the eGFR in this study but rather the ratio of adult circulating testosterone‐to‐estradiol ratio. Some previous studies have reported a significant correlation between the 2D:4D ratio and adult circulating testosterone and estrogen (Muller et al., 2011; Oyeyemi et al., 2018). However, some of these studies were carried out among a clinical population at fertility clinics and as such their findings may have been biased (Manning et al., 1998; Manning et al., 2004). Although some of the studies were performed in a normative population, meta‐analytic studies found no significant relationship between digit ratio and adult circulating hormones (Hönekopp et al., 2007; Klimek et al., 2014; Muller et al., 2011; Oyeyemi et al., 2018). One possible reason for the lack of significant association between 2D:4D and adult circulating testosterone may be that Leydig cells (responsible for testosterone production in males) have different cell populations during the prenatal period, puberty and adulthood. Prenatal Leydig cells degrade after the 24th week of gestation, and the new generation of these cells, which presumably originate from different stem cells, sets on in the postnatal period (Dong et al., 2007; Wu et al., 2007; Zirkin & Papadopoulos, 2018).

The current study has many strengths: Firstly, this study is probably the first to determine the possible interactions between sex and the 2D:4D ratio on the estimated GFR in a healthy adult population; secondly, the 2D:4D ratio was measured using computer‐assisted analysis which is a more precise method than direct measurement (Fink & Manning, 2018); thirdly, the assumptions of linear regression were tested to determine the goodness‐of‐fit of the models of interest. The authors, however, acknowledge that there may be variabilities within a population due to genetic and environmental factors and will therefore recommend further studies in the Ghanaian population.

## 5 CONCLUSION

The estimated glomerular filtration rate was not associated with the 2D:4D ratio but rather with the ratio of the circulating testosterone‐to‐estradiol in a healthy adult population. This may indicate that prenatal hormone exposure may not affect renal function in adulthood. It was also observed that estradiol may have more impact on the eGFR than testosterone although the activity on both on the eGFR is necessary. Further studies are however recommended.

## AUTHORS' CONTRIBUTION

PHY2‐2022‐07‐0301.R1 ‐ Does sex interact with the 2D:4D ratio or adult circulating hormones on the estimated glomerular filtration rate? A cross‐sectional study in Ghana. Moses Bnayeh, Nafiu Amidu: Conceptualization, methodology, project administration and writing – review. Lawrence Obeng, Tuah Kwabena Acheampong, Clement Binwatin Dagungong, Elizabeth Bayor: Methodology, experimentation, data collection and writing – review. Moses Banyeh, Kervin Edinam Zogli: Statistical analysis, results interpretation, writing ‐ original draft and writing – review.

## FUNDING INFORMATION

None.

## CONFLICT OF INTEREST

None.

## Supporting information


Figure S1
