# Supplementary material for: Does sex interact with the 2D:4D ratio or adult circulating hormones on the estimated glomerular filtration rate? A cross‐sectional study in Ghana
Source: Physiol Rep. 2022 Nov 16;10(22):e15516. doi: 10.14814/phy2.15516 (PMC9669620; doi:10.14814/phy2.15516)
Supplement: Supplementary file 1 — Figure S1 [file PHY2-10-e15516-s001.pdf]

Supplementary material

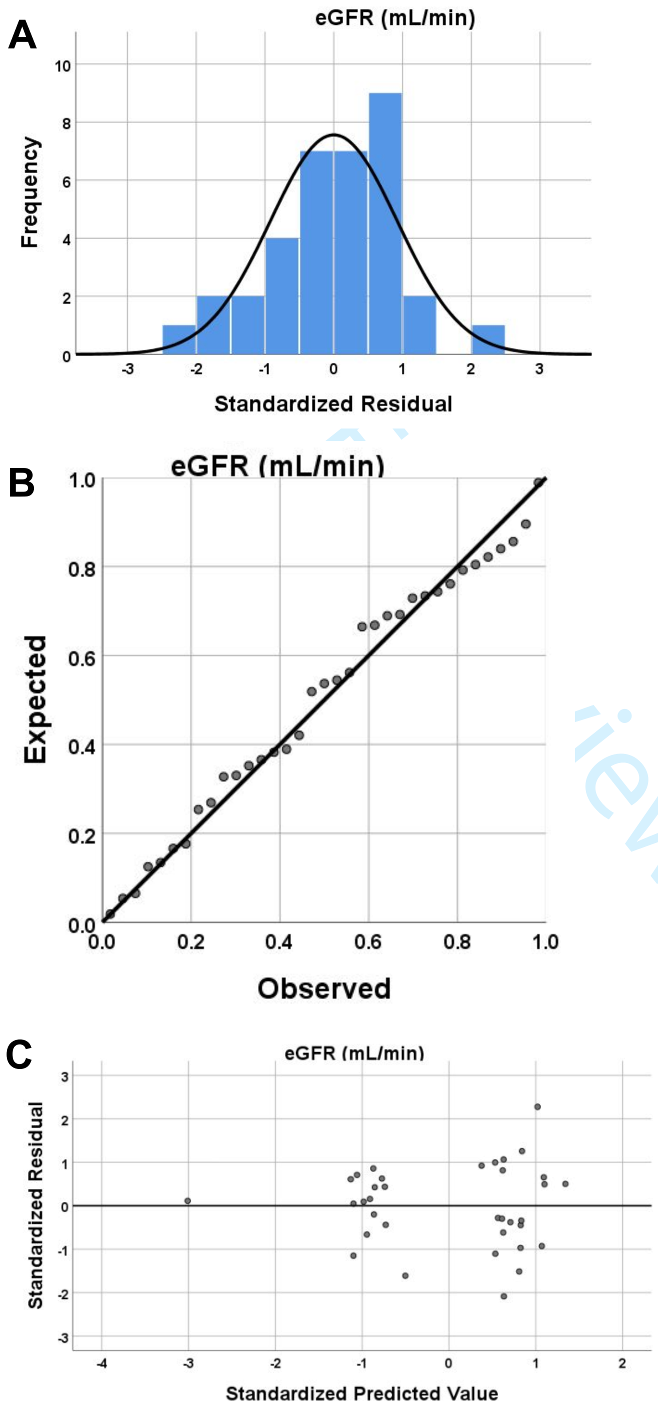

For Review Only

Supplementary Figure S1. The assumptions of multivariable normality and homoscedasticity were tested for LR-8A. The assumption of multivariable normality was tested using histogram (A) and the probability-probability plot (B). The assumption of homoscedasticity was tested using a scatter plot (C).
